# A case of polydactylous nail changes and BAP1-tumor predisposition syndrome: Implications for earlier detection

**DOI:** 10.1016/j.jdcr.2025.11.043

**Published:** 2025-12-10

**Authors:** Lucy Wang, Hasret Gunduz, June Y. Moon, Kenneth Shulman, Mehmet Fatih Atak, Banu Farabi

**Affiliations:** a School of Medicine, New York Medical College, Valhalla, New York; b Elmezzi Graduate School of Molecular Medicine, Northwell Health, Manhasset, New York; c Department of Dermatology, NYC Health + Hospital/Metropolitan, New York, New York; d Department of Dermatology, Icahn School of Medicine at Mount Sinai, New York, New York

**Keywords:** cancer, dermatopathology, genetics, melanocytic nevi, nail disorders, tumour markers

## Introduction

BRCA1-associated protein (BAP1) tumor predisposition syndrome (BAP1-TPDS) is a cancer syndrome caused by loss-of-function variants in the *BAP1* tumor suppressor gene. Although typically acquired in an autosomal dominant pattern, de novo pathogenic variants can also occur, resulting in disease in individuals without an affected parent.^1^ BAP1-TPDS increases susceptibility to various benign and malignant tumors, including BAP1-inactivated melanocytic tumors (BIMTs), uveal melanoma (UM), malignant mesothelioma (MMe), cutaneous melanoma (CM), and renal cell carcinoma (RCC).^2^ Although BIMTs are often the earliest clinical sign of BAP1-TPDS, polydactylous onychopapillomas have recently been identified as another unique diagnostic clue.^3^ We present a patient whose long-standing polydactylous nail changes, suggestive of onychopapillomas, represented a potential opportunity for earlier recognition of BAP1-TPDS.

## Case presentation

**Fig 1:**
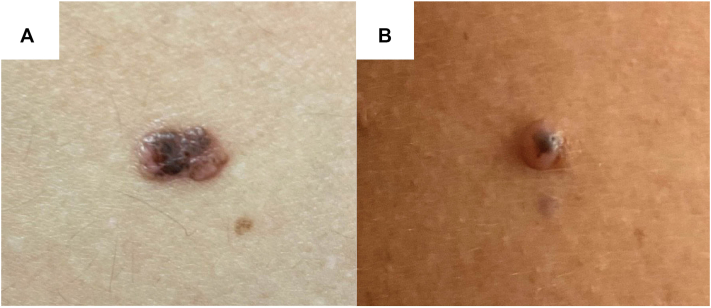
Clinical presentation of BAP1-inactivated melanocytic tumors (BIMTs) on the **(A)** lower back and **(B)** right shoulder.

**Fig 2:**
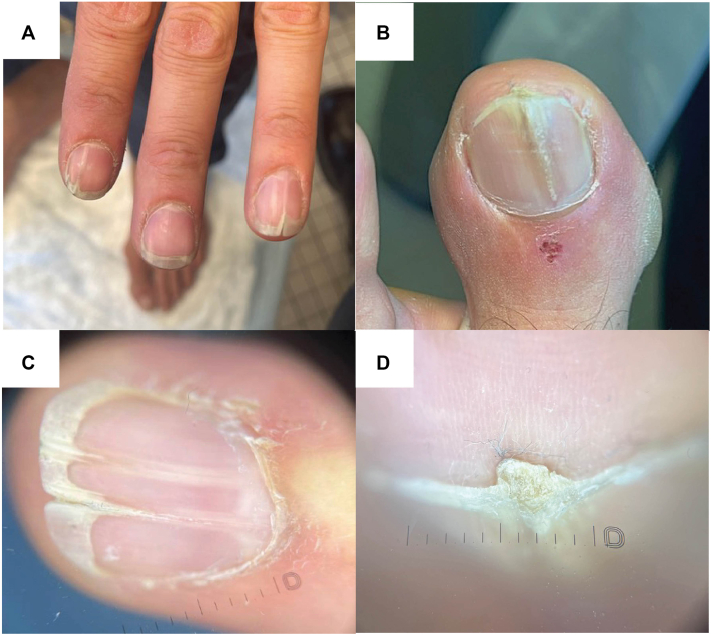
Presentation of polydactylous onychopapillomas. Clinical images of onychopapillomas involving **(A)** multiple fingernails and **(B)** the left first-digit toenail. Dermoscopic images of the **(C)** fingernail with subungual hyperkeratosis, longitudinal leukonychia, and onycholysis, and **(D)** toenail with a subungual hyperkeratotic papule corresponding to overlying longitudinal leukonychia and V-shaped onycholysis.

A 43-year-old male with multiple congenital nevi presented to our clinic for monitoring of an asymmetric 7 mm by 4 mm light-to dark-brown papule on the lower back that had been noticeable for the past 7 years (Fig 1, *A*). He denied any changes or symptoms related to the lesion since the last visit; however, a blue-white veil was observed on dermoscopy, and a shave biopsy was performed to rule out malignant melanoma. A dome-shaped, skin-colored papule with asymmetric black pigment was later found on his right shoulder and biopsied (Fig 1, *B*). Following a total body skin examination, nail abnormalities were noted, which the patient reported having since childhood. Specifically, clinical and dermoscopic examination revealed longitudinal leukonychia, V-shaped onycholysis, distal fissuring, and distal subungual hyperkeratotic papules on multiple fingernails and the left first digit toenail (Fig 2). The clinical presentation of these lesions was strongly suggestive of polydactylous onychopapillomas; however, a biopsy for histopathologic confirmation was deferred while awaiting the results from the skin biopsies.

**Fig 3:**
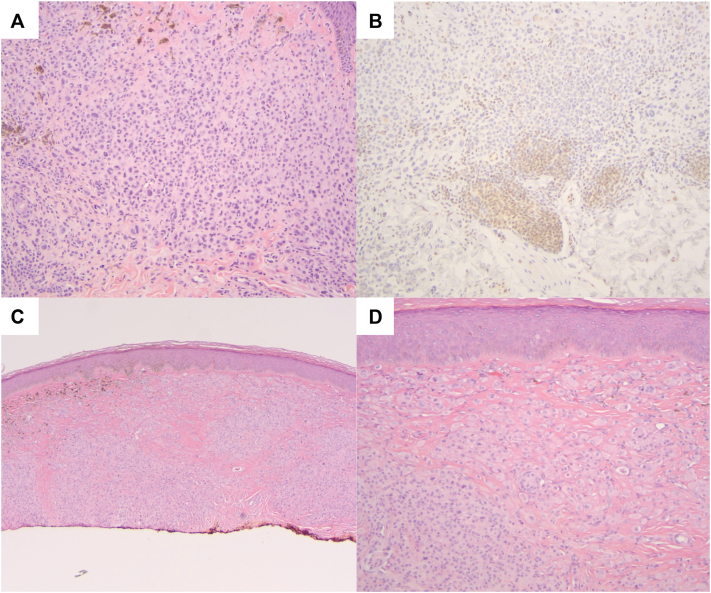
Histopathologic images of the BIMT. **A,** Nodular proliferation of enlarged epithelioid melanocytes with nuclear pleomorphism and amphophilic cytoplasm (H&E, 10×). **B,** BAP1 immunostain demonstrates loss of *BAP1* expression in enlarged epithelioid melanocytes with retained expression in small melanocytes at the base of the lesion (10×). **C,** Nodular intradermal proliferation of enlarged epithelioid melanocytes (H&E, 10×), **(D)** with nuclear pleomorphism and amphophilic cytoplasm (H&E, 20×).

The results of the lower back lesion shave biopsy revealed a BAP1-deficient intradermal melanocytic nevus. Immunohistochemistry (IHC) showed *BAP1* expression in small nevus cells but deficient or absent *BAP1* expression in the larger epithelioid melanocytes (Fig 3). A combined Ki67 and Melan-A stain showed minimal proliferative activity (∼2%), and an HMB45 stain was focally reactive in the superficial component and negative in the majority of the lesion. The biopsy of the right shoulder papule demonstrated a combined melanocytic nevus, consisting of a conventional nevus component and epithelioid melanocytes with loss of BAP1 expression by IHC. The lesions were consistent with BIMTs and completely excised.

The patient was referred to the Genetics Department, and genetic testing with direct sequencing revealed a heterozygous pathogenic loss-of-function variant in *BAP1* exon 4 (NM_004656.4:c.189_198del, p.Thr64Metfs∗5), confirming BAP1-TPDS. Concurrently, a detailed family history was assessed and was not consistent with a hereditary pathogenic variant in the *BAP1* gene, suggesting a *de novo* variant. He was counseled on annual screening for associated malignancies, including ophthalmologic and total-body skin examinations (TBSE), abdominal imaging with ultrasound or magnetic resonance imaging (MRI), and clinical evaluation for MMe or other malignancies.

## Discussion

BAP1-TPDS results from germline pathogenic variants of the *BAP1* gene encoding for the ubiquitin carboxy-terminal hydrolase BAP1. Initially identified for its role in BRCA1 regulation,^4^ BAP1 is now known as an independent tumor suppressor involved in DNA damage repair, cell cycle regulation, and cell growth.^5^ Affected individuals develop CM, UM, MMe, and RCC at a higher frequency and younger age than the general population. Early detection of BAP1-TPDS is crucial as it enables the implementation of targeted cancer surveillance for at-risk individuals. Current recommendations include annual physical examination for symptoms of MMe, TBSE for dermatologic malignancies beginning at age 18-20, and ophthalmologic examinations beginning at age 11-16. Screening for RCC may include annual abdominal ultrasound with biennial MRI or alternating biennial abdominal MRI and ultrasound beginning at age 30.^2^ These screenings maximize early detection and treatment of malignancy. Indeed, identification and treatment of UM when small and localized has been shown to significantly improve prognosis.^6^ The presence of BAP1-TPDS may also inform treatment approach, since studies have suggested worse survival with UM, CM, and RCC, but better survival with MMe when associated with BAP1-TPDS.^2^

As with our patient, individuals often present initially with BIMTs, allowing for BAP1-TPDS evaluation before the development of associated malignancies. These dome-shaped, skin-colored or pink papules typically appear on the head, neck, trunk, and upper extremities. Histologically, BIMTs are intradermal or junctional nevi with BAP1-negative epithelioid dermal melanocytes, making them key indicators for BAP1-TPDS evaluation. However, these lesions often go unbiopsied due to their inconspicuous appearance.

With increasing awareness of their association with BAP1-TPDS, polydactylous onychopapillomas may represent another cutaneous marker for the syndrome.^3^ Onychopapilloma presents as longitudinal erythronychia and distal subungual hyperkeratosis, with other features including longitudinal leukonychia and melanonychia, splinter hemorrhages, onycholysis, and distal fissuring.^7^ While most cases involve a single digit, a recent study found onychopapillomas in 83% (39/47) of BAP1-TPDS patients, with 97% (38/39) showing polydactylous involvement.^3^ Additionally, longitudinal leukonychia was more common than longitudinal erythronychia in BAP1-TPDS, differing from the general population.^3^^,^^7^ In patients with suspected polydactylous onychopapillomas, careful exclusion of additional cutaneous and systemic findings can distinguish them from other conditions that have polydactylous longitudinal leukonychia (eg, Hailey-Hailey disease, tuberous sclerosis complex)^3^ or polydactylous longitudinal erythronychia (eg, Darier disease, lichen planus).^8^ Ultimately, histopathology confirms the diagnosis and should subsequently prompt BAP1-TPDS testing.

Although our patient was diagnosed with BAP1-TPDS following identification of BIMTs, the presence of nail changes highly suggestive of polydactylous onychopapillomas preceding the onset of BIMTs highlights a case in which recognition of this association may have led to earlier detection and surveillance. Thus, we emphasize the importance of the emerging association between polydactylous onychopapillomas and BAP1-TPDS. Clinicians should maintain a high index of suspicion for BAP1-TPDS when polydactylous onychopapillomas are suspected or confirmed, especially in patients with multiple nevi or atypical melanocytic lesions.

## Conflicts of interest

None disclosed.
